# Transmission electron microscope observation of organic–inorganic hybrid thin active layers of light-emitting diodes

**DOI:** 10.1186/1556-276X-7-591

**Published:** 2012-10-25

**Authors:** Yusuke Jitsui, Naoki Ohtani

**Affiliations:** 1 Department of Electronics, Doshisha University, 3-1 Tatara-Miyakodani, Kyotanabe-shi, Kyoto, 610-0321, Japan

**Keywords:** Organic light-emitting diodes, TEM, sol–gel, Hybrid thin films, EDS

## Abstract

We performed transmission electron microscope (TEM) observation of organic–inorganic hybrid thin films fabricated by the sol–gel reaction and used as the active layers of organic light-emitting diodes. The cross-sectional TEM images show that the films consist of a triple-layer structure. To evaluate the composition of these layers, the distribution of atoms in them was measured by energy-dispersive X-ray fluorescence spectroscopy. As a result, most of the organic emissive material, poly(9,9-dioctyl-fluorene-co-*N*-4-butylphenyl-diphenylamine (TFB), was found to be distributed in the middle layer sandwiched by SiO and SiO_2_ layers. The surface SiO layer was fabricated due to the lack of oxygen. This means that the best sol–gel condition was changed due to the TFB doping; thus, the novel best condition should be found.

## Background

Organic light-emitting diodes (OLEDs) have been energetically investigated for application to flat-panel displays and illumination light sources [1-6]. However, the operation lifetime of OLEDs is shorter than that of inorganic LEDs because OLEDs are strictly affected by the oxidant effect. Very recently, we fabricated organic–inorganic hybrid LEDs in which the active layers consisted of organic emissive materials dispersed in SiO_2_[7,8]. These novel LEDs exhibit very long operation lifetime because the organic emissive materials in SiO_2_ are protected against the oxidant effect [9]. However, the structures of the fabricated organic–inorganic hybrid active layers are still unknown. In this research, we observe them by transmission electron microscope (TEM).

## Methods

### Sample fabrication

The organic–inorganic hybrid thin films were fabricated by sol–gel method. Poly(9,9-dioctyl-fluorene-co-*N*-4-butylphenyl-diphenylamine) (TFB) was used as an organic emissive material, while perhydropolysilazane (PHPS) was used as a sol–gel reaction accelerator. The ratio of TFB to PHPS was changed from 1 to 50 wt.%. They were dissolved together in xylene at a density of 3.5 wt.%. Then, a thin film of the TFB-PHPS solution was fabricated on a sufficiently cleaned SiO_2_ substrate by spin-coating method. Next, the samples were annealed to remove the xylene. Finally, the thin film was turned into the organic–inorganic hybrid material by humidity treatment of 90% RH at 50°C for 180 min. This temperature and humidity constituted the best condition for the sol–gel reaction of PHPS recommended by a chemical company, Sanwa Kagaku Corp. (Nagoya, Japan).

### Experimental setup

TEM observation was performed using JEM2100F (JEOL, Tokyo, Japan). To evaluate the distributions of atoms in the organic–inorganic films, energy-dispersive X-ray fluorescence spectroscopy (EDS) observation was additionally performed using JEM2100F. The samples were irradiated using an N_2_ laser for the photoluminescence (PL) measurement. PL spectra were measured using a multi-channel spectroscope (USB-2000, Ocean Optics, Dunedin, FL, USA). PL measurements were performed in atmosphere and at room temperature.

## Results and discussion

**Figure 1:**
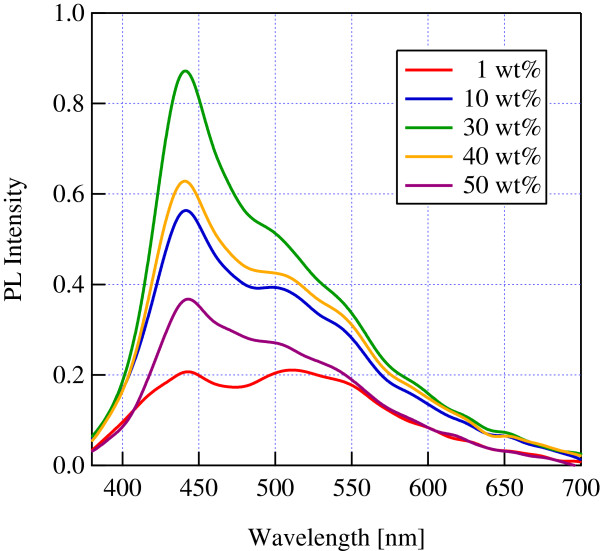
**TFB density dependence on PL spectra.** The density of TFB was changed from 1 to 50 wt.%.

Figure 1 shows the TFB density dependence on the PL spectra of the samples after the sol–gel reaction. All of the PL spectra reveal an emission at around 440 nm, which corresponds to the emission wavelength of TFB before the sol–gel reaction. This means that the light emission mechanism of TFB was not destroyed during the sol–gel reaction. In particular, the brightest emission was observed when the density of TFB was 30%. This is probably because the concentration quenching decreased the emission intensity of TFB when the density was more than 30%.

**Figure 2:**
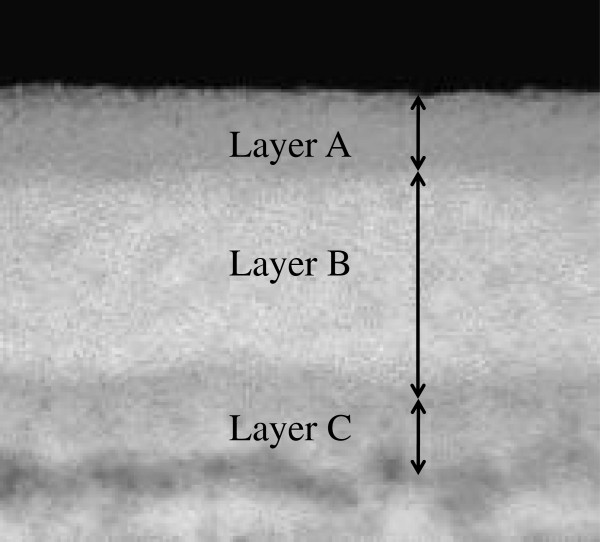
**Cross-sectional TEM image of the organic–inorganic film.** The fabricated thin film was found to be a triple-layer structure.

Figure 2 shows a cross-sectional TEM image of the sample with a TFB density of 30%. It was clearly observed that the fabricated thin film consists of three stacked layers. The thicknesses of these layers from the surface were about 10 nm (Layer A), 30 nm (Layer B), and 10 nm (Layer C). This means that TFB was not uniformly distributed in the fabricated thin film.

**Figure 3:**
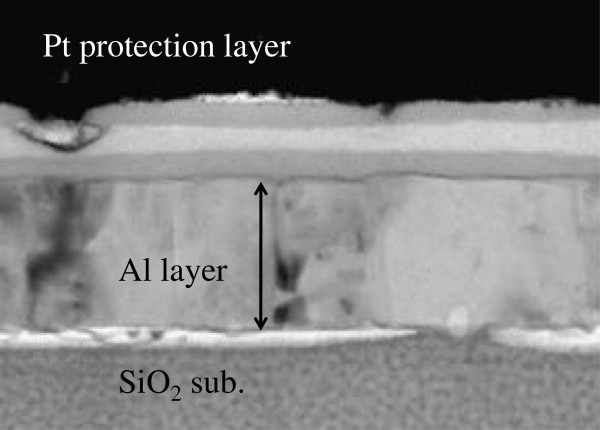
**Cross-sectional TEM image of the organic–inorganic film on aluminum.** To distinguish the fabricated thin film and SiO_2_ substrate clearly, an aluminum layer was deposited on the SiO_2_ substrate. The film fabricated on the aluminum layer clearly reveals a triple-layer structure.

To identify the composition of the layers, the distribution of atoms in them was measured by EDS. Since PHPS was turned into SiO_2_ by the sol–gel reaction, it is difficult to distinguish the fabricated film and the SiO_2_ substrate. Thus, the same sol–gel reaction of PHPS was also performed on aluminum surface. Figure 3 shows a cross-sectional TEM image of the fabricated film on the aluminum surface. The triple-layer structure was clearly observed. This means that the formation of the triple-layer structure does not depend on the substrate.

**Figure 4:**
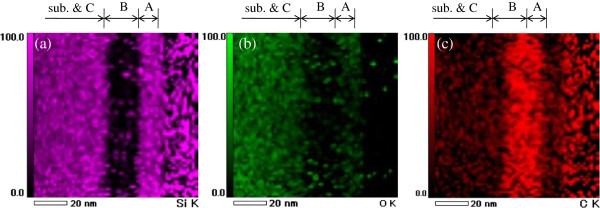
**Cross-sectional images of EDS mapping of the three atoms.** Silicon (**a**), oxygen (**b**), and carbon (**c**).

Figure 4 shows the results of the EDS observation, which clearly reveal the distribution of the three atoms, silicon (Figure 4a), oxygen (Figure 4b), and carbon (Figure 4c). It is very clear that silicon atoms are not contained in Layer B, as shown in Figure 4a. On the other hand, carbon atoms are contained in all three layers, though most are clearly contained in Layer B, as shown in Figure 4c. In addition, few oxygen atoms are found in Layer B, as shown in Figure 4b.

From the results shown in Figure 4, the mass distributions of the three atoms can be identified. Table 1 shows the details of the mass distributions of the three atoms in the three layers, A, B, and C. It is found that the ratio of carbon is largest in all three layers; however, the ratio of carbon is largest in Layer B. On the other hand, the ratio of silicon is smallest in Layer B. This result clearly demonstrates that most of the organic emissive material, TFB, concentrates only in Layer B. Layers A and C consist of silicon and oxygen atoms. However, the ratio of oxygen in Layer A is much smaller than Layer C. Considering the atomic masses of silicon and oxygen, a fine SiO_2_ layer was fabricated in Layer C. On the other hand, the lack of oxygen occurred in Layer A, resulting in the fabrication of undesired SiO. This means that the best condition for the sol–gel reaction, which Sanwa Kagaku Corp. recommends, was changed in the surface of the sample. This is most likely caused by the TFB doping because the best condition for the sol–gel reaction was determined using pure PHPS. Thus, the novel best condition for the sol–gel reaction of TFB-PHPS solution should be investigated. Although the reason for the fabrication of the triple-layer structure is unknown, the question will be solved during the investigation of the best sol–gel condition. Consequently, the organic emissive material TFB was sandwiched by SiO and SiO_2_ layers. We confirmed that the OLED sample contains the same triple-layer structure and works well after soaking in xylene for 1 h. This is because the TFB in Layer B was protected by the SiO layer.

**Table 1:** Mass distributions of the three atoms, carbon, oxygen, and silicon in the three layers

	**Carbon (%)**	**Oxygen (%)**	**Silicon (%)**
Layer A	77.8	7.5	14.5
Layer B	94.5	2.6	0.7
Layer C	76.1	10.7	12.9

## Conclusions

Organic–inorganic hybrid thin films fabricated by sol–gel reaction were investigated in detail by TEM and EDS observations. They consisted of triple-layer structures; most of the organic emissive material TFB was distributed in the middle layer sandwiched by SiO and SiO_2_ layers. The surface SiO layer was formed due to the lack of oxygen. This means that the best sol–gel condition was changed due to the TFB doping; thus, the novel best condition should be found.

## Competing interests

The authors declare that they have no competing interests.

## Authors' contributions

YJ fabricated the samples studied and measured the PL spectra. NO analyzed the results of the TEM and EDS observations and wrote the manuscript. All authors read and approved the final manuscript.
